# Diagnosis and Prevalence (1975–2010) of Sudden Death due to Atlantoaxial Subluxation in Cervical Rheumatoid Arthritis: A Literature Review

**DOI:** 10.1155/2023/6675489

**Published:** 2023-10-05

**Authors:** Eko Agus Subagio, Pandu Wicaksono, Muhammad Faris, Abdul Hafid Bajamal, Diaz Syafrie Abdillah

**Affiliations:** ^1^Department of Neurosurgery, Faculty of Medicine, Universitas Airlangga, Dr. Soetomo General Academic Hospital, Surabaya, Indonesia; ^2^Faculty of Medicine, Universitas Nahdlatul Ulama Surabaya, Surabaya, Indonesia

## Abstract

Rheumatoid arthritis (RA), a chronic inflammatory disease primarily affecting synovial joints and tendons, can potentially impact various organs within the body. One notable complication associated with RA is upper cervical spine instability, medically termed atlantoaxial subluxation (AAS). This condition can lead to adverse consequences, including chronic myelopathy and acute mechanical compression of the medulla oblongata, with the potential for sudden death. While AAS may often remain asymptomatic, some nonspecific symptoms, such as neck pain, have been documented. Severe atlantoaxial subluxation can trigger more distinct symptoms, including delayed occipital pain attributed to the compression of the exiting C2 nerve root. Recent studies have elucidated a spectrum of symptoms preceding sudden death, encompassing vertigo, dizziness, convulsions, dysphagia, disorientation, and seizures. Remarkably, some cases have reported sudden death occurring during sleep. Historical data reveal a fluctuating incidence of this phenomenon, with eleven cases reported between 1969 and 1975 and six cases documented between 1990 and 2010. Notably, one of the most prevalent causes of sudden mortality in individuals with RA is the acute mechanical damage inflicted upon the medulla oblongata due to atlantoaxial subluxation.

## 1. Introduction

Rheumatoid arthritis (RA), a chronic inflammatory disease primarily affecting synovial joints and tendons, potentially impacts various organs within the body. In addition to its peripheral joint manifestations, rheumatoid arthritis has been documented to significantly affect the cardiovascular, pulmonary, and hematologic systems [1]. Notably, there is a lack of precise statistical data on RA patients in Indonesia. However, with a global incidence ranging from 0.5% to 1%, it is estimated that there are likely fewer than 1.3 million individuals with RA in Indonesia [2]. Rheumatoid arthritis frequently leads to cervical spine instability, which can be categorized as atlantoaxial (C1-C2) subluxation (AAS) or subaxial subluxation (C3–C7) (SAS) [3]. The incidence of RA-related cervical spine involvement is substantial, occurring in approximately 36–86% of all RA cases [4].

Atlantoaxial subluxation (AAS) is known as a cause of chronic myelopathy and sudden death due to acute mechanical damage to the medulla oblongata. Patients with RA are reported to have a higher risk of unexpected cardiac mortality, primarily due to an increased prevalence of coronary atherosclerosis when compared to non-RA patients [5]. Sudden death resulting from medulla oblongata compression caused by vertical odontoid process subluxation is rare. Parish et al. documented only 11 cases, with postmortem studies identifying atlantoaxial subluxation as the cause of death. Given the absence of specific symptoms associated with this condition, monitoring and diagnosis may be inadequate [6].

Previous studies have reported that up to 65% of AAS cases worldwide occur in individuals with RA [7], and other studies suggest a range of 40–80% of AAS incidence in rheumatoid arthritis. However, specific data regarding this condition in the context of Indonesia remain scarce [4]. It is worth noting that other causes of AAS include trauma, congenital factors, and inflammation. Notably, inflammation is exclusively attributed to rheumatoid arthritis and represents the primary cause of AAS, while congenital factors are associated with conditions such as Down syndrome (DS). Approximately 30% of DS patients exhibit radiographic evidence of AAS, but only 1% of them experience symptomatic AAS [8].

Similar to the pathophysiological processes observed in RA affecting other peripheral joints, cervical RA triggers inflammation within the synovial joints of the cervical spine. This inflammation leads to the development of pannus, resulting in erosion of the juxta-articular bones. Ultimately, these processes detrimentally impact the articular structures and result in swelling of the associated ligaments. The atlantoaxial joints (C1-C2), which are most frequently affected by RA within the cervical spine, possess an exceptionally wide range of motion compared with other vertebrae. Consequently, a damaged atlantoaxial joint is susceptible to various pressures and strains, potentially leading to anterior, posterior, lateral, rotational, or vertical subluxation [4, 9].

## 2. Prevalence of Rheumatoid Arthritis

The prevalence of rheumatoid arthritis (RA) is estimated to affect approximately 0.1–2.0% of the global population. According to the 2014-2015 National Health Survey, Australia reported the highest prevalence of RA among the countries studied. Among Native American populations, Pima and Chippewa Indians exhibited the highest prevalence of RA, with rates of 5.3% and 6.8%, respectively. In contrast, rural areas in South Africa showed a low prevalence of RA at 0.0026% and rural areas in Nigeria reported zero prevalence of RA [10].

In Indonesia, though the precise number of individuals with RA remains unclear, it is estimated that fewer than 1.3 million out of the country's 268 million people in 2020 suffered from RA. Data from Indonesia indicate that in Central Java, the prevalence of RA was 0.34%, while in Malang, the prevalence of RA was 0.5% in the Municipality area and 0.6% in the Regency area among individuals over 40 years of age. RA prevalence and incidence exhibit variations among different populations, with women being 2-3 times more likely to develop RA than men. Although the incidence may increase with age, there is no statistically significant difference reported between women and men over 70 years of age. The highest incidence of RA is reported in the age group of 50–54 years [2].

## 3. Prevalence of C1C2 Subluxations and Anatomic Abnormalities

A cohort study conducted in Japan reported 238 cases of cervical spinal cord compression events associated with C1-C2 subluxations. Another study found that among 200 patients, 66 individuals (33%) exhibited high-riding vertebral artery (HRVA), while 90 patients (45%) had narrowed pedicles, as identified through thin-sliced pedicular-oriented CT (TPCT) scans [11]. In a meta-analysis study, the prevalence of spinal cord compression was found to be 2.3%. However, the specific location within the cervical spine was not specified [12].

In the results of a study conducted by Le Quellec in 2022, it was noted that out of a total of 323 rheumatoid arthritis patients with atlantoaxial subluxation, the majority were in the age range of 45–50 years. The duration of the disease varied, with some individuals having RA for 2 years, 5 years, or even more than 10 years. A disease duration of more than 10 years was identified as a significant predictor of cervical spine damage [13]. Nevertheless, it is important to note that atlantoaxial subluxation can occur at any age, and age alone is not a barrier to its development in rheumatoid arthritis. Regarding gender distribution, atlantoaxial subluxation occurs relatively evenly among both males and females. However, in individuals without predisposing factors, atlantoaxial instability is exceptionally rare [8].

## 4. Atlantoaxial Subluxation: Symptoms and Signs

While AAS may often remain asymptomatic, some nonspecific symptoms, such as neck pain, have been documented. Severe AAS can result in delayed occipital pain, attributed to the compression of the exiting C2 nerve root. Additionally, individuals with AAS may exhibit a loss of cervical lordosis and experience resistance to voluntary spinal movement. Lateral subluxation and rotation can cause tilting of the head. The most severe symptoms arise when cervical spinal cord impingement occurs. These symptoms include weakness and sensory disturbances in the upper and lower extremities, as well as sphincter dysfunction, leading to urinary retention and incontinence, and even sudden death. Examinations in such cases may reveal long tract symptoms associated with myelopathy, such as extensor plantar reflexes, clonus, positive Hoffman's sign, and bilateral motor weakness with increased tone and spasticity. It is crucial to note that due to the extensive peripheral joint damage caused by arthritis and the potential sarcopenia accompanying long-standing active RA, the debility induced by myelopathy can be easily overlooked. Signs and symptoms of spinal cord compression tend to be progressive once they manifest [9]. In Mikulowski's 1975 study, symptoms preceding sudden death were mentioned, including vertigo, dizziness, convulsions, dysphagia, disorientation, and seizures. Some individuals even experienced falling asleep before suddenly passing away [14].

## 5. Diagnostic Evaluation of Spinal Cord Compression due to C1-2 Subluxation in Cervical Rheumatoid Arthritis (RA)

Given the critical importance of preventing sudden death resulting from C1-C2 subluxation in cervical rheumatoid arthritis (RA), a comprehensive understanding of proper diagnostic evaluation for early detection is critical. Considering that the majority of patients do not exhibit significant symptoms of cervical instability, readily accessible and cost-effective diagnostic tools such as typical anterior/posterior, lateral, and open-mouth radiographs, along with dynamic lateral flexion/extension images, may suffice for early detection. The flexion/extension view is essential due to the documented limitations of a standard static lateral projection, which has shown missed detections of atlantoaxial instability (AAI), an underestimation of its severity, and inadequate assessment of recovery. Several metrics can be employed when assessing plain radiographs for cervical instability, aiming to ascertain the presence and severity of the condition.

**Figure 1:**
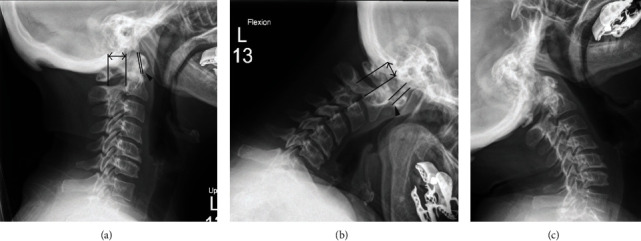
(a) Lateral radiographs of a patient with AAI. In the neutral view, the AAI (arrowhead) is 1 mm. (b) In flexion, the AAI increases to 7 mm. (c) In extension, the AAI reduces to their neutral measures [1].

**Figure 2:**
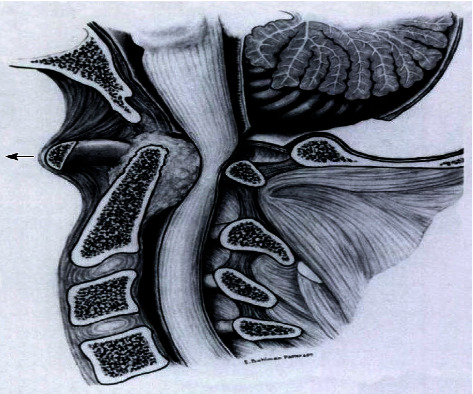
Illustration of pannus on the cervical [15].

In the evaluation of AAI, it is necessary to measure the anterior and posterior atlantodental intervals. The anterior atlantodental interval is defined as the distance less than three millimeters between the posterior arch of C1 and the anterior boundary of the socket visible on the transverse axis of C1. It is important to note that AAI exhibits a 3 mm gap and is not fixed by flexion and extension, as it is elevated by flexion and can be lowered by extension (Figure 1). To provide an angle for reference, a maximum AAI limitation range of 6–10 mm has been proposed. This limitation is crucial because a larger AAI is associated with a higher risk of spinal cord compression, and an AAI exceeding 5 mm indicates clinically significant AAS instability [7]. It is important to be aware that AAI has limitations when applied to individuals with cranial settling (CS) [1].  Figure 2 illustrates subluxation of the anterior atlas in the axis, the formation of a pannus around the odontoid process, and bone degradation. Notably, severe compression of the spinal cord occurs between the anterior pannus and the posterior atlas arch [15]. Observing the presence of pannus in the early stages offers the potential to prevent adverse effects. A case report study in 2020 noted that pannus could disappear after immobilizing the atlantoaxial segment. Furthermore, there was no evidence of its migration to other areas or upward spreading, even over a five-year follow-up period, despite the systemic disease remaining unchanged [4].

Previous studies have consistently identified rheumatoid arthritis (RA) with cervical myelopathy as a significant contributor to neurological disorders [16]. In the most severe cases, this condition can even result in sudden death. It is crucial to recognize that delaying the surgical procedure may lead to the development of myelopathy [17].

## 6. Sudden Death Prevalence on C1C2 RA

Sudden death in individuals with atlantoaxial subluxation due to rheumatoid arthritis (RA) is indeed a rare occurrence, and recent cases documented in the literature over the past decade are challenging to find, as reflected in Table 1. As a consequence, recent meta-analyses continue to rely on dated sources, specifically studies conducted in the 1960s, for their references. In Mikulowski's retrospective study from 1975, it was observed that the mortality rate was higher among patients with cervical RA and atlantoaxial subluxation (AAS) compared with those without AAS. Among the 52 deaths recorded, three were attributed to atlantoaxial subluxation in RA, while the rest were due to other causes [14].

Mikulowski's study further detailed 11 cases from 1969 to 1974. Three of these cases were asymptomatic, while two exhibited an additional diagnosis, namely, cerebral thrombosis, accompanied by atypical leg pain. Distinguishing between cerebral thrombosis and atlantoaxial subluxation in the context of cervical RA was found to be challenging. One patient experienced vertigo after initiating indomethacin medication, followed by a month of clonic cramping and confusion. Case 8 experienced a sequence of vomiting followed by widespread clonic cramping for two months before eventual demise, and notably, no Babinski sign was observed. In the ninth case, a humerus fracture was reported, followed by the onset of mental disorders one month prior to death. The last case presented a history of swallowing difficulties persisting for several months, along with voice changes [14]. Subsequent autopsies conducted in instances of sudden death frequently revealed the development of rheumatoid inflammatory pannus. This pannus formation often results in compression of the spinal cord and other nervous tissues, primarily due to the instability of the cervical spine [17].

In a 2015 meta-analysis, a decreased prevalence of atlantoaxial subluxation in the 2000s was observed in comparison to the pre-1980s period (24% versus 36%) [18]. This finding aligns with the clinical experience of spinal surgeons, who noted a decline in the rate of occipital-cervical fusion, coinciding with the introduction of more potent curative antirheumatic drugs (DMARDs) and treatment strategies. However, it is worth noting that despite a decrease observed in the 1980s, Ward's study in California did not find a significant reduction in deaths due to atlantoaxial subluxation in rheumatoid arthritis patients between 1983 and 2001. The Neva study conducted in Finland in 2001, which examined one-year cervical spine radiographs from 33 patients, identified severe cervical spine deformities in 17 patients, which had the potential to cause fatal complications. Among these cases, four resulted in sudden death [17]. The decrease in sudden death rates due to atlantoaxial subluxation in rheumatoid arthritis may also be attributed to the infrequent practice of routinely conducting autopsies, often due to technical difficulties [19].

In Gerges's study in 2022, it was found that 50% of the total patient sample died from atlantoaxial subluxation in cervical rheumatoid arthritis with myelopathy [17]. In another study by Janssen in 2020, 2.5% of rheumatoid arthritis patients reported cervical myelopathy. In such cases, atlantoaxial subluxation was identified as the primary cause of spinal cord and medulla oblongata compression, which was followed by the formation of pannus [20].

Postmortem investigations have reported that 10% of cervical rheumatoid arthritis patients exhibited cranial settling formation due to pannus, resulting in spinal cord compression and sudden death. Cranial settling is a condition that leads to myelopathy, significantly elevating the mortality risk associated with it. The prospects for successfully addressing instability through decompression and stabilization treatment become limited once myelopathy has set in because compression has already affected the medulla oblongata [4]. Sudden death resulting from medulla oblongata compression involves several mechanisms, including dysphagia, dysarthria, and those associated with the vagus, glossopharyngeal, and hypoglossal nerve compression [20].

## 7. Predictive Factors for Sudden Death in Cervical Rheumatoid

Numerous risk factors associated with the development of cervical subluxation have been identified, and some of these factors are linked to more severe underlying medical conditions [21]. However, it is important to note that there may be variations or occasional contradictions in the correlations observed between studies. These discrepancies can arise from differences in the groups and cohorts studied, as well as unaccounted confounding factors. In a comprehensive review and meta-analysis conducted in 2017, which limited its analysis to moderate-to-high quality studies, the following factors were found to be associated with increased likelihood of cervical spine instability [21–23]:

1. Female
2. Positive rheumatoid factor (RF)
3. Long-term glucocorticoid use
4. Long RA duration
5. Peripheral arthritic joint disease (in hands or feet)
6. Increased erythrocyte sedimentation rate and C-reactive protein levels
7. Previous joint surgery
8. Hand, foot, hip, and knee erosions, as well as rapidly progressing erosive peripheral joint disorders
9. Early lateral joint subluxations
10. Osteoporosis
11. Degree of myelopathy, as shown in Table 2.

## 8. Conclusion

Atlantoaxial subluxation in rheumatoid arthritis is indeed a rare but potentially fatal occurrence that can lead to sudden death. Historical data reveal that between 1969 and 1975, 11 cases were reported, while studies spanning from 1990 to 2010 reported an additional 6 cases. It is essential not to underestimate the gravity of this condition, as many cases go undiagnosed until they culminate in sudden death. One of the most significant findings in these cases is that the primary causes of sudden death resulting from atlantoaxial subluxation in rheumatoid arthritis are acute mechanical damage to the medulla oblongata and persistent compression myelopathy. Moreover, the absence of postmortem evaluations (autopsies) conducted on cases of sudden death associated with atlantoaxial subluxation in rheumatoid arthritis underscores the urgency of addressing this condition and the need for further investigation and understanding.

## Figures and Tables

**Table 1:** Literature summary.

Author/year	Country	Methods	Number of cases	Postmortem evaluation
Mikulowski et al., 1975	Sweden	Case postmortem with autopsy	10104 case RA with AAI, 11 sudden death (1969–1975)	All case compression medulla
Parish et al., 1990	Georgia	Case report	1 RA case with AAI sudden death	Compression medulla
Neva et al., 2001	Finland	Case study in one year in Finland	17 cases with AAI, 4 case RA with AAI sudden death	Compression medulla
Unuma et al., 2010	Japan	Case report	1 RA case with AAI sudden death	Compression medulla

**Table 2:** Cervical rheumatoid myelopathy Ranawat classification [21].

Class I	Normal neurologically
Class II	Subjective weakness with hyperreflexia and dysesthesia
Class IIIa	Objective weakness with long track sign but ambulatory
Class IIIb	Objective weakness with disability to walk or feed oneself, quadriparesis

## Data Availability

Previously reported (such as case report, literature review, etc.) data have been used to support this study and are available at (DOI or OTHER PERSISTENT IDENTIFIER). These previous studies (and datasets) are cited as references at the appropriate places in the text.
